# Shiga Toxin-Producing *Escherichia coli* O157 Shedding Dynamics in an Australian Beef Herd

**DOI:** 10.3389/fvets.2017.00200

**Published:** 2017-11-27

**Authors:** Christina Ahlstrom, Petra Muellner, Geraldine Lammers, Meghan Jones, Sophie Octavia, Ruiting Lan, Jane Heller

**Affiliations:** ^1^Epi-interactive, Wellington, New Zealand; ^2^School of Animal and Veterinary Science, Charles Sturt University, Wagga, NSW, Australia; ^3^School of Biotechnology and Biomolecular Sciences, University of New South Wales, Sydney, NSW, Australia

**Keywords:** *Escherichia coli*, molecular epidemiology, food safety, MLVA typing, Shiga toxin-producing *Escherichia coli* O157

## Abstract

Shiga toxin-producing *Escherichia coli* (STEC) O157 is an important foodborne pathogen that can be transmitted to humans both directly and indirectly from the feces of beef cattle, its primary reservoir. Numerous studies have investigated the shedding dynamics of *E. coli* O157 by beef cattle; however, the spatiotemporal trends of shedding are still not well understood. Molecular tools can increase the resolution through the use of strain typing to explore transmission dynamics within and between herds and identify strain-specific characteristics that may influence pathogenicity and spread. Previously, the shedding dynamics and molecular diversity, through the use of multilocus variable number of tandem repeat analysis (MLVA) of STEC O157, were separately investigated in an Australian beef herd over a 9-month study period. Variation in shedding was observed over time, and 33 MLVA types were identified. The study presented here combines the two datasets previously published with an aim to clarify the relationship between epidemiological variables and strain types. Three major genetic clusters (GCs) were identified that were significantly associated with the location of the cattle in different paddocks. No significant association between GCs and individual cow was observed. Results from this molecular epidemiological study provide evidence for herd-level clonal replacement over time that may have been triggered by movement to a new paddock. In conclusion, this study has provided further insight into STEC O157 shedding dynamics and pathogen transmission. Knowledge gaps remain regarding the relationship of strain types and the shedding dynamics of STEC O157 by beef cattle that could be further clarified through the use of whole-genome sequencing.

## Introduction

Ruminants are the primary reservoir of Shiga toxin-producing *Escherichia coli* (STEC) O157 (1), a significant foodborne pathogen that has the potential to cause disease in humans (2). STEC infections can have severe clinical outcomes, including death in young children (3). An estimated 2,801,000 global STEC O157 acute human illnesses occur annually (4), and in the US, 68% of STEC O157 infections are considered to be foodborne (5). Ground beef or fertilizer derived from cattle effluent is implicated as the vehicle in many foodborne outbreaks (6, 7) and sporadic illnesses (8), prompting large scale control efforts to minimize STEC contamination along the production chain in the USA (9) and Australia (10, 11). Environmental and animal contact are also considered significant exposure pathways for sporadic STEC O157 human infections (12). Shedding of STEC O157 by beef cattle has been described both in feedlot and pasture based settings (13), with variation in prevalence estimates between herds and between regions (10, 14, 15). Super shedding has been implicated as a significant driver of pathogen transmission (16, 17); however, its role in the epidemiology and transmission dynamics of *E. coli* O157 is still being explored (18).

The molecular epidemiology of STEC O157 in cattle is complex, as multiple subtypes are often circulating simultaneously on individual farms (19). Further, identical subtypes can be found on farms separated by hundreds of kilometers without any evidence of an epidemiological link between farms (9). Traditionally, molecular typing of STEC O157 has primarily been based on pulse field gel electrophoresis, multilocus variable number of tandem repeat analysis (MLVA), or phage typing (20–22). However, these comparative typing methods have shortcomings that may be exacerbated when supported by insufficient epidemiological study designs (23).

With the increased application of whole-genome sequencing technology in outbreak investigations (24), progress is being made in better capturing the evolutionary and transmission dynamics of STEC O157. For example, different lineages have been associated with human disease and asymptomatic carriage in the bovine host and genomic signatures have been identified that are associated with altered pathogenic potential (25). The identification of phylogenetically informative single nucleotide polymorphisms (SNPs) has further supported epidemiological investigations of STEC O157 transmission, offering discriminatory power unmatched by macrogenomic techniques (26, 27).

A better understanding of the temporal variation of STEC O157 shedding can help target control efforts. Strain-specific characteristics, such as pathogenicity and adaptation mechanisms, can influence shedding (28) and ultimately control strategies (16). Thus, the application of molecular tools in a spatiotemporal context can greatly enhance the understanding of infectious disease dynamics (29–31). For example, persistence of a single strain type versus shedding of multiple types over time may be an indicator for different epidemiological patterns, exposures, and transmission routes.

To address the challenges described, the shedding dynamics and molecular diversity of STEC O157 in an Australian beef herd was recently investigated (32, 33). Variation in shedding was observed both within and between cattle over time, with a total of 33 MLVA profiles identified during the course of the 9-month longitudinal study. Clonal replacement was found to occur over time as well as shedding of identical strains between cows. The objective of the work presented here was to integrate epidemiological data with molecular typing results from said studies to further analyze the spatial and temporal trends in genotypes shed and to better understand the previously identified patterns of shedding synchronization.

## Materials and Methods

### Sample Collection, Bacterial Culture, and Strain Typing

As described in Lammers et al. (33), a beef herd of 23 Hereford cattle was sampled either one or two times per week between 4th October 2012 and 20th June 2013, resulting in a total of 58 sampling points. Cattle were grazed on a total of seven different paddocks, varying between 3.5 and 7 ha in area, according to the availability of grass, herd nutritional requirements, and environmental management considerations. During the study period, the cows had calves at foot until weaning in January 2013 and a bull joined the herd in October 2012. Apart from these changes, there was no contact with other livestock. Areas grazed had not been grazed by other livestock for months before the study, and no manure had been spread. Fecal samples were collected from each cow by rectal palpation or during defecation using a new disposable sleeve glove. Gloves were individually placed in sterile Whirl-Pak bags (Nasco, Australia) and stored on ice until arrival at the laboratory. The use of animals in the study was previously approved by Charles Sturt University Animal Care and Ethics Committee Protocol number 12/060.

Fecal samples were processed as described previously (33). Briefly, 10 g of feces was homogenized in 90 ml of sterile buffered peptone water (Oxoid, Australia). Before incubation at 37°C for 18–24 h, 100 µl was plated onto sorbitol MacConkey + 5-bromo-4-chloro-3-indolyl-b-d-glucuronide agar (Oxoid) (CT-SMAC + BCIG) containing cefixime (0.05 mg/l) and potassium tellurite (2.5 mg/l) (Oxoid). Plates were screened for non-sorbitol fermenting and β-glucuronidase-negative colonies and up to 10 presumptive colonies were tested using an *E. coli* O157 latex test (Oxoid). Samples negative after direct plating were subjected to immunomagnetic separation, plated onto CT-SMAC + BCIG, and incubated for 18–24 h at 37°C. Up to 10 individual colonies were confirmed as STEC O157 using a multiplex PCR, targeting the *rfbE, stx_1_*, and *stx_2_* genes (34). Standard biosecurity and safety procedures were carried out in line with institutional processes.

MLVA typing of eight VNTR loci was performed on a total of 168 confirmed *E. coli* O157 isolates previously by Jones et al. (32), using the method described in Hyvtiä-Trees et al. (35). The fragment size of PCR products representing eight separate loci was determined using an automated ABI3730 DNA analyzer (Applied Biosystems), and the number of repeats was calculated using known data for the reference strain EDL933.

Eighteen of the 168 isolates were sequenced by Jones et al. (32) using the MiSeq sequencing platform (Illumina, San Diego, CA, USA), and previously identified SNPs (32) were used for this study. The 18 isolates were selected to represent eight distinct MLVA types (MTs), including types appearing throughout the study period, and representing isolates from the same MLVA type from different cows and from the same cow over time. The WGS data were used to set cutoff values for MLVA genetic clusters (GCs) as detailed below.

### Combined Data Analysis

A bootstrapped minimum spanning tree (MST) based on MLVA profiles was created using the software MSTgold v2.4 (36). Default parameters were used, with a run time of 1 h. Null alleles (i.e., no PCR amplification) were treated as missing data. One hundred bootstrap pseudo-replications were performed to establish confidence levels. Distances were calculated on the basis of the difference in the numerical values of the alleles, rather than all alleles being equidistant. The number of possible unique MSTs was calculated using both methods; however, the number of possible trees was much smaller using the difference method, with an estimated 902 (±46) and 49,270 (±6,790) possible MSTs for the difference and equidistant method, respectively.

Previous WGS analysis by Jones et al. (32) revealed that two isolates with up to five VNTR locus differences could still be nearly identical by WGS. GCs in the current analysis were, therefore, assigned based on the number of locus differences between these two MLVA types (MT16 and MT25) in the MST. Four MLVA types were detected along the most likely chain of evolution between MT16 and MT25, as determined by the highest supported MST, with each of those MLVA types differing by a maximum of two loci. Therefore, MLVA types of identical isolates based on WGS were considered to belong to the same GC as well as any MLVA type that differed by a maximum of two loci from any other MLVA type within that cluster.

The genetic dataset indicating STEC O157 GCs was merged with metadata from Lammers et al. (33). Fisher’s exact tests were performed to determine if GC was independent of cowID and paddock number. The number of isolates belonging to each GC detected throughout the study period was visualized in a bar chart created using the package ggplot2 (37) in the statistical program R v3.2.4 (38).

## Results

**Figure 1:**
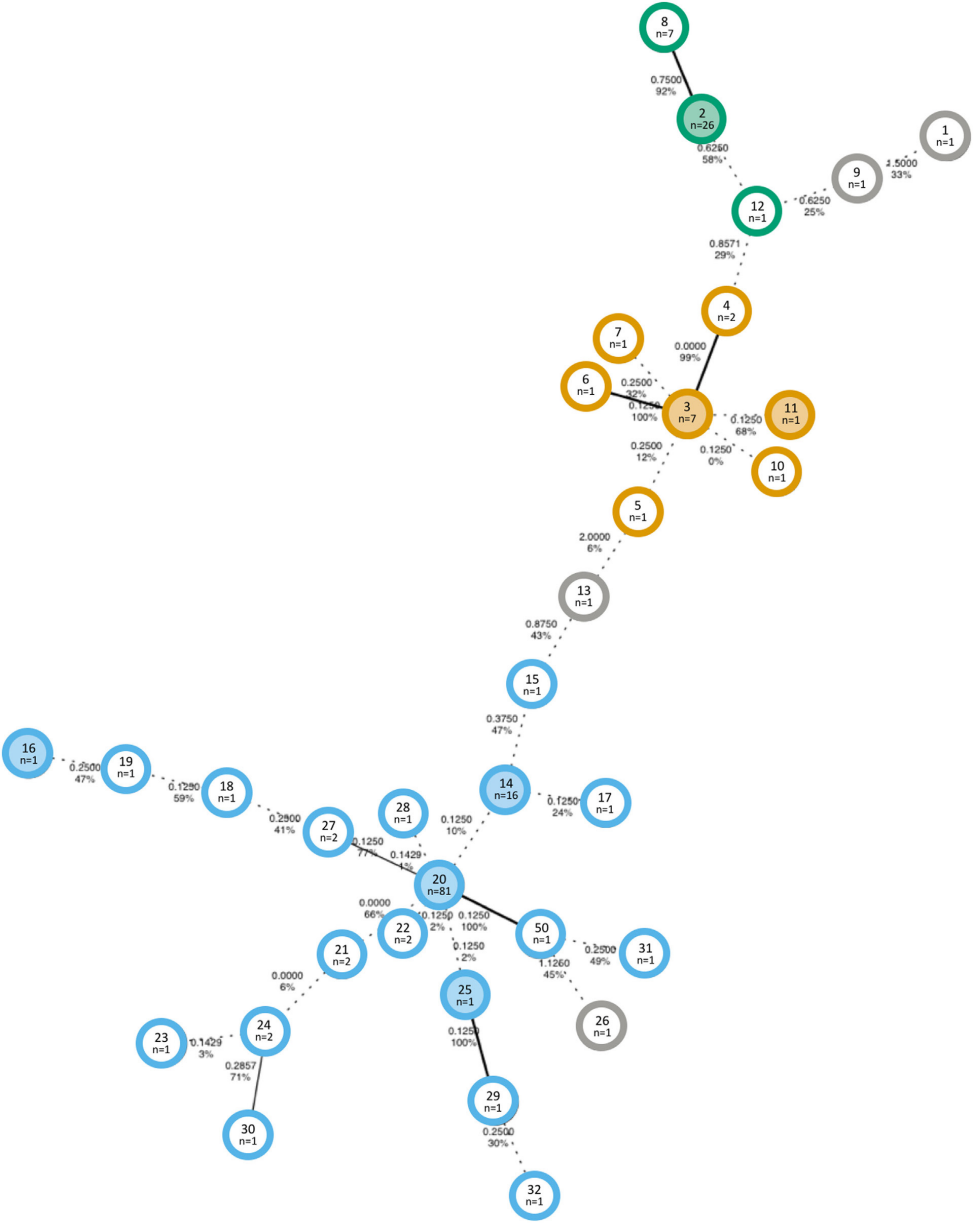
Minimum spanning tree with the highest bootstrap value of Shiga toxin-producing *Escherichia coli* O157 isolates collected in an Australian beef herd over a period of 9 months. The distances represent the arithmetic pairwise distance of alleles at eight VNTR loci and are shown on the connecting edges of the tree. Bootstrap support values are also indicated. Edges with less than 70% bootstrap support are depicted by dashed lines. Colors indicate the genetic cluster (GC): GC1 = blue, GC2 = orange, GC3 = green, and unclustered = grey; and shaded nodes indicate that at least one isolate of that MLVA type was analyzed by whole-genome sequencing. The text in the center of each node displays the MLST type (top) with the number of isolates of that type (bottom).

Previously reported molecular typing data from Jones et al. (32), including MLVA types and WGS results, were used for this study. To analyze the spatial and temporal trends of the MLVA genotypes, we used a new approach to define relationships of MLVA types and GCs. We first computed the relationship of MLVA types that had WGS data and determined a VNTR difference of two loci as the cutoff for GCs. Using this cutoff, we used bootstrapped MSTs to estimate the relationship between 33 different MLVA types. The MST with the highest bootstrap percent (43 ± 5.8) is composed of 33 nodes, each representing a unique MLVA type (Figure 1). A total of three GCs were identified based on a maximum distance of two loci to be considered the same cluster. A consensus network MST was also computed (not shown), which supported the GC definitions obtained from the bootstrapped MST. Nineteen, seven, and three MLVA types were found in GC1, GC2, and GC3, respectively. Four MLVA types (1, 9, 13, and 26) did not belong to a GC, as they differed by at least three locus variants from any other type.

MT14, MT2, and MT3 were each the first shed MLVA type of GC1, GC2, and GC3, respectively, with MT2 and MT3 each found at a central node in the MST in relation to the remaining members of the cluster. MT14 was the first GC1 MT to be isolated (on 5th November 2012) and was predominantly found in the beginning of the study period, whereas MT20 was the most commonly isolated MLVA type and the only GC1 type found after movement to the final paddock on February 28, 21 weeks after the start of the study.

**Figure 2:**
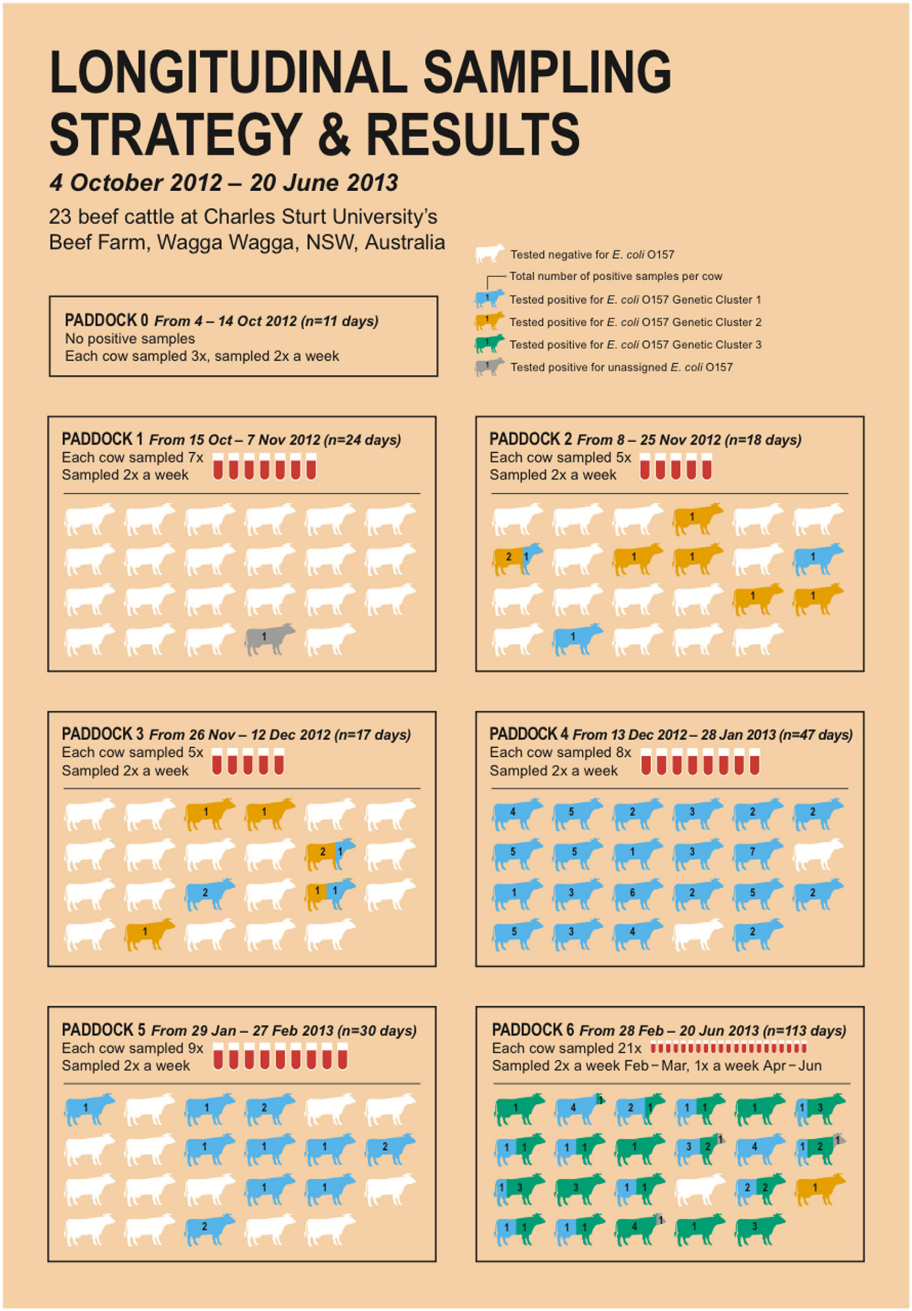
Infographic illustrating the sampling strategy and results from a 9-month longitudinal study. The number of cows sampled and the number of samples collected per paddock are shown. The number of positive samples for Shiga toxin-producing *Escherichia coli* (STEC) O157 per cow per paddock is indicated by numbers, and colors indicate the STEC O157 genetic cluster (GC): GC1 = blue, GC2 = orange, GC3 = green, unclustered = gray, and no STEC O157 positive samples = white.

All cows shed isolates from at least one of the GCs during the 9-month study period, with 9% (*n* = 2) shedding isolates from a single GC, 61% (*n* = 14) shedding isolates from two different GCs, and 30% (*n* = 7) shedding isolates from three different GCs (Figure 2). Five cows shed isolates from a previously isolated GC after shedding a different GC (e.g., shed Cluster 1, then Cluster 2, then Cluster 1 again). Further, in six instances, an isolate from a different GC was recovered in consecutive sampling points. Figure 2 illustrates the number of GCs isolated from each cow in each paddock. Cows spent the most amount of time in Paddock 6, which is also the only paddock where all three GCs were found. A significant difference was found in the distribution of GCs between paddocks (*P* < 0.001), but not within cows (*P* = 0.142), based on Fisher’s exact tests.

**Figure 3:**
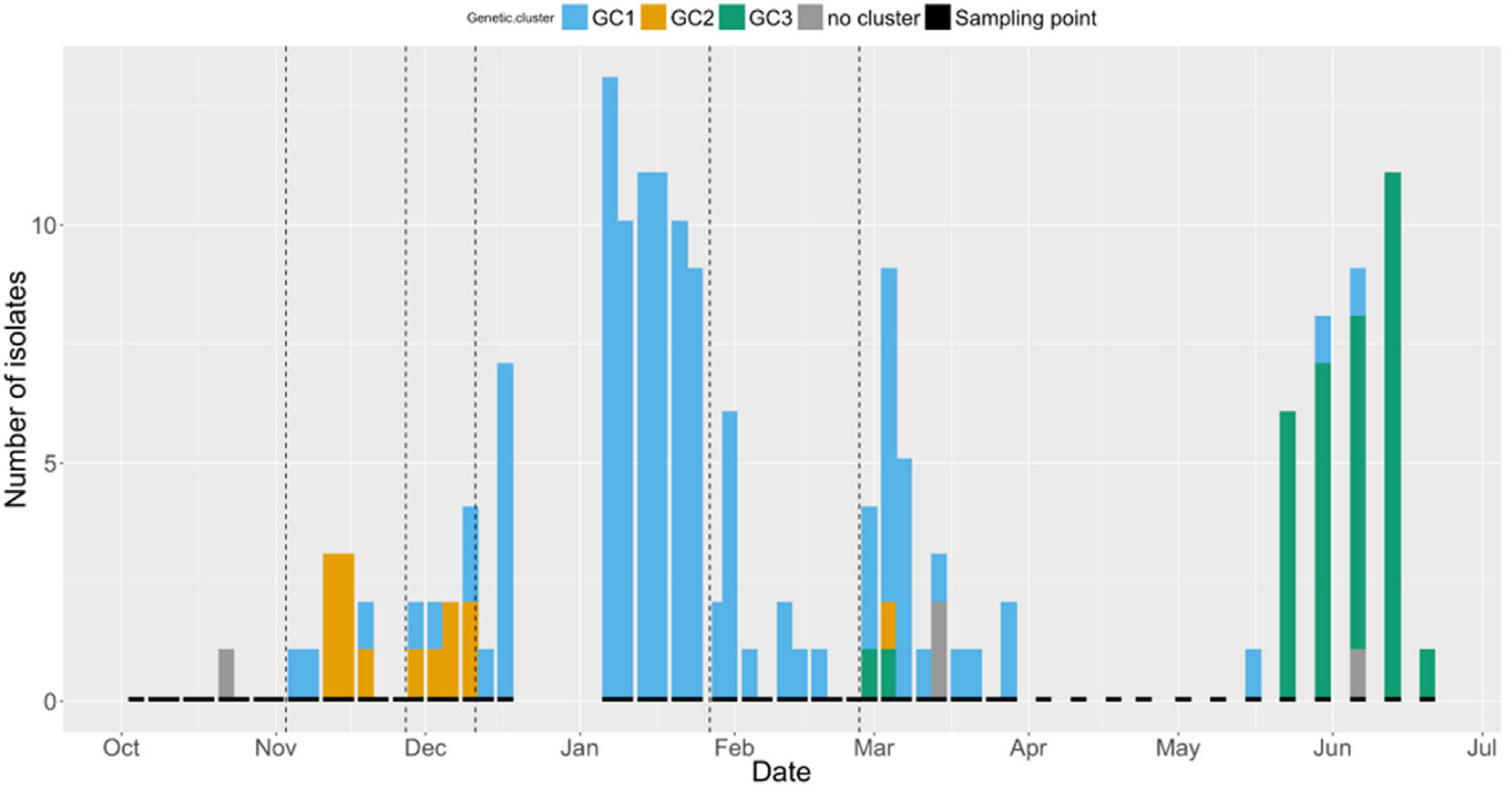
Bar chart displaying the number of Shiga toxin-producing *Escherichia coli* O157 isolates at each sampling point (indicated by short black bars) between 4th October 2012 and 20th June 2013. Colors indicate the genetic cluster (GC): GC1 = blue, GC2 = orange, GC3 = green, and unclustered = gray. Dashed vertical lines indicate change of paddock.

The number of isolates identified from each GC at each sampling point is shown in Figure 3. A trend can be seen, with a higher number of GC2 isolates found in the early part of the study period, followed by an apparently bimodal distribution of GC1 shedding in the middle, and increasing numbers of cows shedding GC3 isolates at the end of the study period. GC1 was isolated from cows in all paddocks, with the exception of Paddock 1. GC2 was the most frequently isolated GC in Paddock 2 and 3 but was not detected after cattle were moved to Paddock 4. GC3 was only isolated from cattle in Paddock 6.

## Discussion

The analysis presented here expands upon previous results from a 9-month longitudinal study of 23 co-grazed beef cattle in Australia (32, 33), supporting a comprehensive investigation of the shedding dynamics of STEC O157 genotypes. Three STEC O157 GCs were detected, as well as four unique MLVA types that did not belong to any of the GCs identified. Most cows shed MLVA types from more than one cluster. The investigation revealed a stronger association between GC and paddock number than between GC and individual cow ID, which supports the hypothesis that shedding of *E. coli* strain types is more strongly influenced by environmental exposure, than individual cow attributes (39).

Interestingly, the number of cows shedding GC1 isolates appeared to follow a bimodal epidemic curve, with the first peak occurring in mid-January and the second in early March. Toward the end of the study period, GC3 showed a similar trend with an increasing number of cows shedding that cluster until the study terminated in June. This might indicate that cows developed an immune response to the different GCs, resulting in a peak shedding period, followed by a period of decreased shedding. This is supported by the fact that despite not showing clinical signs of illness, cattle do mount an immune response to STEC O157 (40) and decreased shedding has previously been observed in cows vaccinated against STEC O157 (41, 42), with evidence of strain dependence (43).

Co-colonization of individual cows with multiple genotypes has been documented previously (44) and is likely to have occurred here. Shedding of a previously isolated GC after shedding of different GCs suggests possible co-colonization, which was observed in five cows over the course of the study period. However, coinfection with multiple genotypes cannot be confirmed, as only one isolate was saved from each cow per sampling point.

Interestingly, a change from Paddock 3 to 4 and a change from Paddock 5 to 6 marked noticeable shifts in the STEC O157 population structure identified in this analysis. In the first instance, GC2 seemed to disappear from cattle upon changing to a new paddock. Conversely, GC3 was not detected until cattle moved to Paddock 6. This again suggests that environmental forces may play a substantial role in the colonization of cattle, such as movement to an environment where new STEC O157 types are present or the introduction of a new type (e.g., by wildlife vectors). For example, starlings have been implicated in the transmission of STEC O157 between dairy farms (45), and free-ranging deer sharing rangeland with cattle have been found to shed STEC O157 (46). Isolation and genetic analysis of STEC O157 from the environment and wildlife populations in this study would help elucidate the sources and transmission dynamics between different hosts and environments.

The use of the distance method to generate an MST was found to be the most appropriate for the data available, as single-repeat changes were previously observed to occur most frequently (47); however, in that same study, 25% of the mutational events included multiple-repeat changes. Such large changes would likely not have affected the assignment of GCs in this study as, by definition, GC was inclusive of single-locus variants. Here, the definition of GC was supported by WGS data of 18 isolates representing eight MLVA types with genome lineages 1, 2, and 3 corresponding to the GC1, GC2, and GC3, respectively. Consequently, genotypes were clustered into groups differing by no more than two loci, which is the same threshold previously suggested by Mellor et al. (48). Previous analysis of the same MLVA data by Jones et al. (32) used a threshold of a single VNTR difference to define a clonal complex; however, this conflicts with the WGS data that showed that isolates with as many as five VNTR differences were closely related. Thus, WGS provides necessary resolution to infer relatedness of strains in this context and should be incorporated into the interpretation of other typing methods when such data are available.

While the results generated by this study have provided additional insight into STEC O157 transmission dynamics on the study site, there are some shortcomings. In this study, paddock changes were convenience based and driven by herd management needs, such as pasture quality and nutritional requirements, and thus cows did not spend equal amounts of time in each location. As such, additional analysis of STEC O157 shedding patterns in other herds and environments are needed to substantiate our findings. Furthermore, as with any epidemiological study, unaccounted for selection bias could have occurred. The strong sampling design did, however, offer insight into the spatiotemporal nature of STEC O157 shedding of cattle in this pasture based environment.

This study used WGS data to assist defining MLVA GCs. In the absence of WGS information, the true evolutionary relationship of isolates within and between MLVA types cannot be confirmed. Care should be taken when making inferences about the population dynamics based on MLVA data, and results should be considered to be of preliminary nature. This is highlighted by the fact that isolates with up to five VNTR locus differences can still be nearly identical based on WGS. This is not entirely surprising, as Vogler et al. (47) determined that the mutation rate of VNTR loci in STEC O157 is high and variable between loci, confounding the analytical power of MLVA. Similarly, MLVA typing of *Mycobacterium avium* subspecies *paratuberculosis* was found to be unreliable to detect related and unrelated isolates, as it both over- and underestimated relatedness in comparison with WGS (49).

Importantly, results from the current analysis could inform potential future typing studies that were out of scope for the investigation presented here and could provide a rationale for the prioritization of additional isolates for WGS using existing samples. For example, building onto these initial studies, STEC O157 isolates (*n* = 295) from an intensive sampling study performed by the same research group (50) have been collected and could be used to investigate the substantial diurnal variation in shedding that was identified in 24 cows sampled one or two times per day for 2 weeks. Such high-resolution genetic analyses would provide further insight into STEC O157 daily shedding dynamics in Australian beef cattle if based on molecular typing methods compatible with the spatiotemporal range under investigation. We suggest that MLVA typing in isolation would not be appropriate in this context and that WGS would be needed to resolve daily trends in STEC O157 shedding.

In conclusion, this study has provided further insight into STEC O157 shedding dynamics in an Australian beef herd over time and identified environmental forces as a likely driver of pathogen transmission. The knowledge gained can be used as a stepping stone to design higher resolution studies to answer additional questions related to cow- and herd-level shedding synchronization of specific strains over time.

## Author Contributions

PM, CA, and JH drafted the manuscript and made a lead contribution to the conception, design, analysis, and interpretation of the work. GL, MJ, SO, and RL critically reviewed the manuscript and made contributions to field, laboratory, analysis, and interpretation of the work.

## Conflict of Interest Statement

The authors declare that the research was conducted in the absence of any commercial or financial relationships that could be construed as a potential conflict of interest.
